# Enhancement of Polymeric Immunoglobulin Receptor Transcytosis by Biparatopic VHH

**DOI:** 10.1371/journal.pone.0026299

**Published:** 2011-10-14

**Authors:** Chris D. Emmerson, Els J. van der Vlist, Myrthe R. Braam, Peter Vanlandschoot, Pascal Merchiers, Hans J. W. de Haard, C. Theo Verrips, Paul M. P. van Bergen en Henegouwen, Edward Dolk

**Affiliations:** 1 Biomolecular Imaging, Department of Biology, Faculty of Science, Utrecht University, Utrecht, The Netherlands; 2 Cell Biology, Department of Biology, Faculty of Science, Utrecht University, Utrecht, The Netherlands; 3 Ablynx NV, Zwijnaarde, Belgium; Tulane University, United States of America

## Abstract

The polymeric immunoglobulin receptor (pIgR) ensures the transport of dimeric immunoglobulin A (dIgA) and pentameric immunoglobulin M (pIgM) across epithelia to the mucosal layer of for example the intestines and the lungs via transcytosis. Per day the human pIgR mediates the excretion of 2 to 5 grams of dIgA into the mucosa of luminal organs. This system could prove useful for therapies aiming at excretion of compounds into the mucosa. Here we investigated the use of the variable domain of camelid derived heavy chain only antibodies, also known as VHHs or Nanobodies®, targeting the human pIgR, as a transport system across epithelial cells. We show that VHHs directed against the human pIgR are able to bind the receptor with high affinity (∼1 nM) and that they compete with the natural ligand, dIgA. In a transcytosis assay both native and phage-bound VHH were only able to get across polarized MDCK cells that express the human pIgR gene in a basolateral to apical fashion. Indicating that the VHHs are able to translocate across epithelia and to take along large particles of cargo. Furthermore, by making multivalent VHHs we were able to enhance the transport of the compounds both in a MDCK-hpIgR and Caco-2 cell system, probably by inducing receptor clustering. These results show that VHHs can be used as a carrier system to exploit the human pIgR transcytotic system and that multivalent compounds are able to significantly enhance the transport across epithelial monolayers.

## Introduction

All cavities of the human body are lined by epithelial tissue. Epithelia are generally a single layer of cells which are connected by junctions to form a barrier between the inside of the body and lumina. Epithelial cells are polarized, in that they have a segregated plasma membrane (apical and basolateral membrane) and a partly segregated endosomal system (e.g. basolateral early endosomes and apical recycling endosomes). Although epithelia serve as a barrier, they do have the ability to specifically transport molecules across via several means. One way is the transport pathway called transcytosis, which is a receptor mediated vesicular transport route that connects the apical and basolateral sides of the cell, thereby giving the body a way to selectively take up and secrete molecules [1]. Several receptors have been described to be able to transport molecules across epithelia via the transcytotic transport route. One of these receptors is the polymeric immunoglobulin receptor, which is able to transport dimeric immunoglobulin A (dIgA) and to a lesser extent pentameric immunoglobulin M (pIgM) across epithelial cells [2]. After synthesis, the pIgR is delivered to the basolateral membrane [3], [4], from where it internalizes either with or without bound dIgA/pIgM and subsequently moves via several transport itineraries to the apical membrane [5]. A covalent interaction via disulfide bridges will form en route between the pIgR and dIgA/pIgM in the case the receptor has bound a ligand [6]. At the apical membrane a large part of the pIgR ectodomain is cleaved off by (an) as of yet unknown protease(s), giving rise to the compound known as the secretory component. The secretory component is thereby secreted, either with or without the dIgA/pIgM into the mucosa [7], [8]. Due to the covalent interaction, the secretory component will remain attached to the dIgA, a compound referred to as ‘secretory IgA’ (sIgA). Through this interaction it gives the immunoglobulin more stability in the mucosa [9]. By transporting immunoglobulins across the epithelia of the intestines and lungs, the pIgR transcytotic system ensures humoral defense in the mucosa against incoming pathogens.

Although the fate of the pIgR in trafficking might seem unidirectional, a small percentage of pIgR present on the apical membrane remains uncleaved and this population of receptors has the ability to internalize again [10], [11]. In the case a ligand is bound to the receptor, swift recycling will occur to the apical membrane. However, in case no ligand is bound, the receptor has the ability to transcytose back to the basolateral membrane. A classic example here, is the observation made a few years ago that *Streptococcus pneumoniae* is able to make use of this latter transport pathway to gain entry into the body [12], [13]. So, although the main transport vector of the receptor is towards the apical membrane, there is also a small vector in the opposite direction.

The ability to traffic in both the basolateral-to-apical and apical-to-basolateral direction makes the pIgR an interesting therapeutic target that could mediate secretion of unwanted compounds out of the body or mediate uptake of orally administered therapeutic compounds into the body. Several groups have already published studies of proteins which are able to bind to and transcytose with the pIgR (Fab-fragments [14] and 9 amino acid peptides [15]). Here, we have used VHH technology as a therapeutic approach, since this platform has several advantages over the aforementioned compounds. VHHs, therapeutically known as Nanobodies®, are the isolated variable domains of heavy chain-only antibodies derived from camelids [16]. They are small (∼15 kD) and have been recognized in literature for their therapeutic potential because of their high chemical and physical stability, relatively low production costs, and cleft binding capabilities. In addition, the simple gene make-up of VHHs lends them well for genetic cross linking of several VHHs in order to make multidomain compounds. This makes optimization of the molecule easy because either the affinity for the target can be increased by linking two VHHs which bind the same protein (multivalency) or multispecific proteins can be created by linking two VHHs with different effector functions [17].

In this study we have performed phage display selections on VHH libraries constructed from *Llama glama* immunised with hpIgR ectodomain to select for compounds which can mediate epithelial transcytosis. We show that the selected VHHs have high affinity for the receptor and are able to transcytose with the receptor across a monolayer of MDCK cells transduced with the hpIgR gene and Caco-2 cells which endogenously express the hpIgR. Furthermore, by making multivalent constructs, we were able to increase the initial rate of entry and also the transcytotic rate of one such compound, probably by inducing receptor clustering on the basolateral membrane. Our data show that the selected and optimised VHHs against the ectodomain of hpIgR are a promising tool for unidirectional transport of cargo across epithelial monolayers.

## Results

### Selection and validation of hpIgR binding VHHs

**Figure 1:**
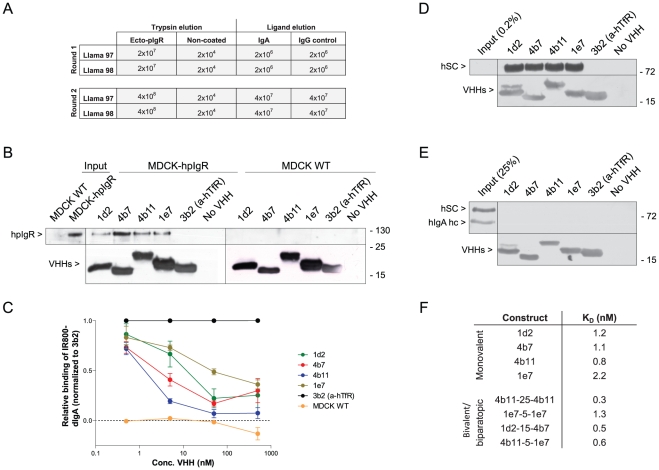
Selection and validation of VHHs against hpIgR. (A) Outputs of phage display selection on coated recombinant hpIgR ectodomain with either trypsin or IgA elution for llama's 97 and 98. (B) Pull down of VHHs with incubated cell lysate of MDCK control (right) or MDCK-hpIgR (left) cells with the anti-his matrix Talon. Western blot with anti-hSC and anti-myc tag (VHH) (n = 3). (C) A critical amount of IR800 labelled dIgA with indicated amount of VHHs was incubated on MDCK-hpIgR cells at 4°C for 1.5 h. The normalized amount of IR800-dIgA is calculated and set out in the graph against the VHH concentration (n = 4) (s.e.m.'s are shown for all points). (D+E) Pull down of VHHs incubated in MDCK-hpIgR cultured medium containing hSC (D) or with 4 µg/ml sIgA (E). Western blot with anti-hSC, anti-hIgA heavy chain and anti-myc (n = 4). (F) VHHs were titrated and put on cells for 1.5 h at 4°C. VHH presence was quantified by using anti-myc tag and D-α-M-IR800 antibodies. Quantification as described in experimental procedures. K_D_ determinations were done with a ‘one-site specific binding’ algorithm in Graphpad. (n = 6).

In order to obtain a source of VHHs that are able to bind to the human polymeric immunoglobulin receptor (hpIgR), two *Llama glama's* were immunized with the recombinant ectodomain of hpIgR. After five boosts with similar amounts of antigen, a blood sample was taken and analysed for immune reactivity on a pIgR coated plate, which indicated pIgR reactive antibodies. Lymphocytes were subsequently extracted and a phage display library was made. With these immune libraries two successive rounds of selection were done on coated hpIgR ectodomain. Bound phages were eluted using trypsin (total bound phages). The first round yielded approximately 2×10^7^ total bound phages for both libraries (fig. 1A), which was enriched in comparison with a non-coated control (2×10^4^ phages). In order to select for VHHs binding to the dIgA binding epitopes on pIgR, IgA was used to compete in the re-binding of released phages, thereby specifically selecting those VHHs that bind the pIgR on the IgA binding site. When phages were eluted with IgA, 2×10^6^ phages were released from the coated plate. The control, IgG eluted well, yielded similar amounts of eluted phage as the wells eluted with IgA. The output of the IgA eluted wells was used for another round of selection in a similar manner as the first round to further enrich for pIgR binding VHHs. Here, a total of 4×10^8^ bound phages were eluted with trypsin for both libraries (with 2×10^4^ phages for the non-coated control) and 4×10^7^ phages were eluted with IgA which in both cases (trypsin and IgA) was higher than the first round whilst input was roughly the same, indicating an enrichment of hpIgR binding VHHs.

Monoclonal VHHs were picked out of a polyclonal output and tested for their ability to bind coated hpIgR ectodomain. Of the positive, sequentially unique, monoclonal VHHs, four clones were selected on the basis of their promising binding characteristics. These four VHHs were only able to pull down the hpIgR from a hpIgR transduced MDCK cell lysate and not from a MDCK control lysate (fig. 1B), whilst a negative control VHH directed against the human transferrin receptor was not able to pull down the hpIgR.

Specificity was furthermore tested by analysing competition of the four VHHs with the IR800 labelled physiological ligand, dIgA (fig. 1C). All four VHHs were able to compete to different extents with dIgA for binding to coated hpIgR ectodomain. IC_50_ values of the constructs ranged from ∼1 nM (4b11) to ∼70 nM (1e7). In accordance with this latter feature, these four VHHs were able to pull down secretory component (hSC) from the cultured medium of polarized MDCK-hpIgR cells (fig. 1D), whilst purified secretory IgA (sIgA) could not be precipitated by these VHHs (fig. 1E), indicating that the covalent interaction between dIgA and hSC blocks the binding site of the VHHs.

Finally, the affinity of the VHHs was determined by compound titration on live cells. VHHs were titrated and allowed to bind non-polarized MDCK-hpIgR cells at 4°C. Quantitation of the bound VHHs yielded sigmoid curves typical for binding assays. B_max_ was roughly the same for all compounds, however there were differences in the concentration at which half-maximal binding was observed, which is a measure of affinity. Plotting with a ‘one-site specific binding’ algorithm yielded K_D_ values for the four VHHs (fig. 1F). Of the selected VHHs, 4b11 appears to bind strongest to the receptor, having a K_D_ value of roughly 0.8 nM. The other VHHs seem to have affinities between 1 and 2 nM. Together these data indicate that these VHHs are all able to bind specifically and with high affinity to the unoccupied hpIgR.

### Selected VHHs internalize into cells expressing hpIgR

**Figure 2:**
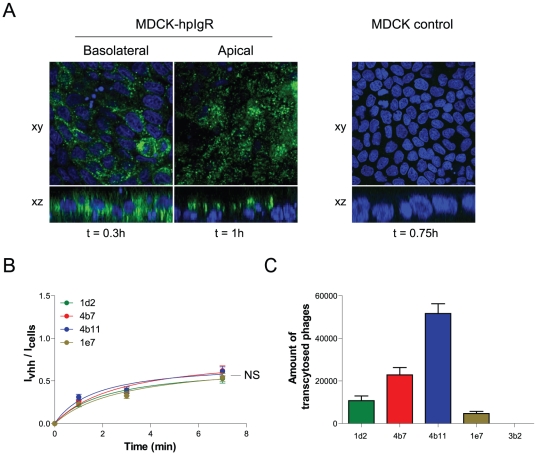
Selected VHHs internalize into and transcytose across polarized epithelial cells expressing hpIgR. (A) 1 µM of 4b7 VHH was applied either basolaterally (for 20 min) or apically (for 60 min) on polarized MDCK-hpIgR cells and visualized after fixation with an anti-myc tag and G-α-M-Alexa488 antibody and DAPI staining both in xy and xz plane (n = 4). 1 µM of 4b7 VHH was applied to confluent MDCK control cells and treated as above (n = 4). (B) Internalization assay of selected VHHs. 1 µM VHH was incubated on cells at 37°C for 1, 3 or 7 min. Externally bound VHH was removed with 25 µg/ml trypsin for 45 min on ice. Internalized VHH was immunolabelled with an anti-myc tag and D-α-M-IR800 antibodies. Quantification of fluorescence is described in experimental procedures. Fluorescence intensities of the internalized amount is divided over the amount of cells (To-pro3 nuclear stain) and plotted against time. The signal of the irrelevant anti-hTfR VHH was deducted from the specific VHH signal and therefore represents the x-axis (n = 12) (s.e.m.'s are shown for all points). (C) Transcytosis assay using VHH displaying phages. Phages were applied basolaterally and incubated for 5 h. Amount of phages in apical medium is subsequently quantified as described in experimental procedures and plotted per construct (n = 4) (s.e.m.'s are shown for all points). Significance measurements were done by two-way ANOVA.

To see whether these VHHs are able to internalize into polarized MDCK cells in a hpIgR dependent fashion, MDCK cells expressing hpIgR were grown on transwell inserts. Paracellular leakage was analysed using lucifer yellow and was found to be negligible after 5 days of culturing. 1 µM of the four selected VHHs was applied either 20 min basolaterally or 60 min apically on the polarized MDCK cells at 37°C. Cells were subsequently fixed and internalised VHH was immunostained with anti-myc tag antibodies and analysed by confocal microscopy. All four anti-hpIgR VHHs were able to internalize into polarized MDCK-hpIgR cells from both the basolateral and, albeit more poorly, apical side (fig. 2A; only VHH 4b7 is depicted, however staining was similar for all VHHs). No internalization of the VHHs was seen in control MDCK cells that do not harbour hpIgR (fig. 2A). VHH staining in MDCK-hpIgR cells showed puncta which partly colocalized with the early endosomal marker EEA1, affirming that VHHs enter the endocytic system of MDCK cells after internalization (fig. S1; again only 4b7 is shown).

Next, internalization rates of the four VHHs were determined. VHHs were diluted to 1 µM and applied to non-polarized MDCK-hpIgR cells for varying times at 37°C. Internalization was stopped by putting the cells on ice. The VHHs still present on the outside surface were removed by incubating the cells on ice with 25 µg/ml trypsin for 45 min. This protocol was beforehand shown to be sufficient to remove any surface bound VHH (data not shown). Internalized VHH was subsequently immunolabelled with an infrared dye (IR-800) and quantitated as described in the experimental procedures. Data are presented as the intensity of internalized VHH over the intensity of total cells present in the wells as determined by a fluorescent nuclear dye (I_vhh_/I_cells_). This in order to correct for any variation in the number of cells used in the experiments (fig. 2B). An irrelevant VHH which specifically recognizes human transferrin receptor and therefore serves as an indicator of fluid-phase uptake was used as a negative control. The internalized amount of the irrelevant VHH was deducted from all time points of the anti-hpIgR VHHs. Our data show that all VHHs are actively taken up by these cells already at early time points. The signal was seen to reach a plateau after ∼8 min of internalization time, possibly indicating that the receptor recycles in non-polarized cells. The internalization rates of the four VHHs are similar, probably reflecting constitutive internalization of the receptor.

To test whether these VHHs have the potential to form a carrier for therapeutic use, the constructs were assessed on their transcytotic capacity. The VHHs were expressed on bacteriophages and used in transwell assays with polarized MDCK-hpIgR cells. Phages were applied basolaterally and incubated for 5 hours with the cells. The apical medium was subsequently harvested and used to infect TG-1 bacteria to estimate the number of phages transcytosed over 5 hours (fig. 2C). Phage input was chosen at that concentration where the anti-hTfR VHH did not show any transport across the polarized cells. At this concentration, specific VHHs displayed different transcytotic capacities, 4b11 giving the most transcytosed phages (mean = 10.320±447) and 1e7 the least (mean = 937,6±198) per hour. Apically added phages were not able to significantly transcytose to the basolateral chamber (data not shown). These data indicate that the selected anti-hpIgR VHHs are able to cross an epithelial monolayer from the basolateral to the apical side to varying degrees and are able to take along cargo (the virion) during this transport.

### Optimization of transcytotic capacity

**Figure 3:**
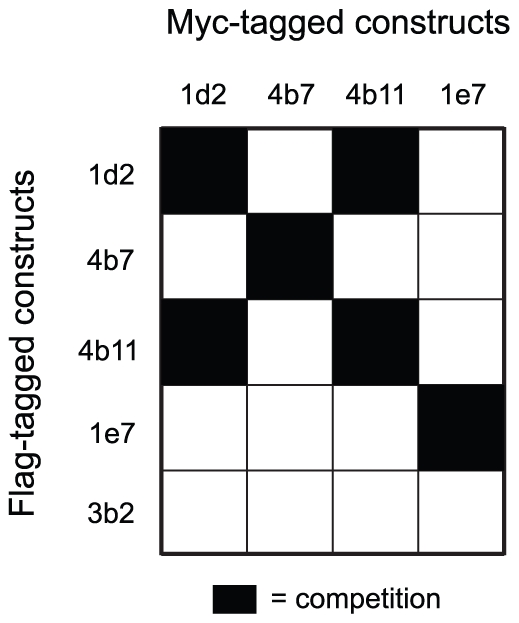
Epitope mapping of VHHs. Myc-tagged VHHs were incubated with increasing amounts of flag-tagged VHHs in an hpIgR coated well for 2 h at room temperature. Myc tag presence was subsequently quantified and competition therewith assessed. Competition is depicted in a chequer board diagram were black indicates competing VHHs (n = 2). See Figure S2 for raw data.

Recently it has been shown that the clustering of receptors induces or augments their endocytosis, for example in the case of the transferrin receptor [18] or the epidermal growth factor receptor [19]. Specifically for the pIgR it has been suggested that upon binding, dIgA induces pIgR dimerization and this feature enhances pIgR endocytosis [20]. We hypothesized that a bivalent construct, a construct with two identical heads genetically coupled together, could induce dimerization of the pIgR and that a biparatopic construct, a construct with two heads targeting two different, non-overlapping, epitopes, could induce dimerization and/or oligomerization of the receptor on the plasma membrane. To test if the VHHs recognized different non-overlapping epitopes on the hpIgR, epitope mapping of the selected VHHs was first undertaken by means of competition assays. VHHs were produced with either a myc- or flag-tag. Increasing concentrations of the flag-tagged constructs were mixed with a critical amount of the respective myc-tagged constructs and allowed to bind to a hpIgR coated plate. After binding, myc-tag presence was quantified in the wells. The flag version of each VHH was able to compete off the corresponding myc version at increasing concentrations in all cases, thereby validating the assay (fig. 3 and S2). Competition could also be seen in the case of 1d2 and 4b11 at increasing concentrations of the other compound, which indicates that they bind to the same area on the receptor. The other two VHHs appear to have unique binding sites as no other compound could give competition except itself. So, within this set of selected VHHs there appear to be three epitope groups despite the fact that all VHHs compete with dIgA: the 1d2/4b11 epitope, the 4b7 epitope and the 1e7 epitope.

**Figure 4:**
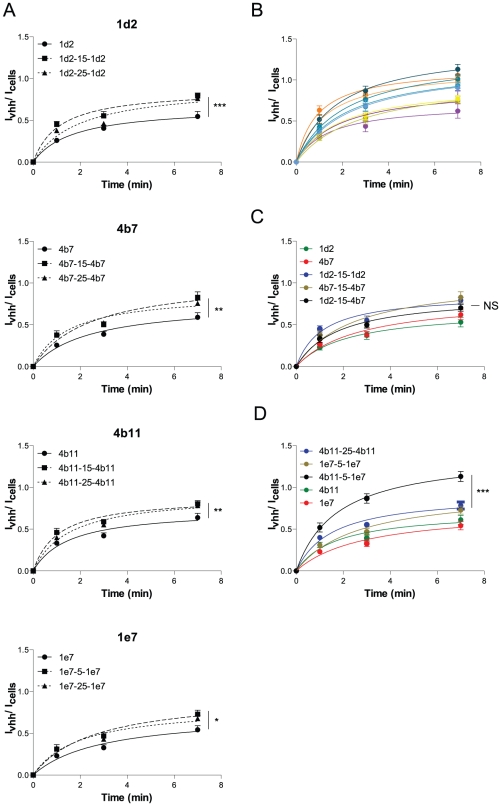
Bivalency enhances internalization. (A–D) Internalization assay of selected VHHs. 1 µM VHH was incubated on cells at 37°C for 1, 3 or 7 min. Externally bound VHH was removed with 25 µg/ml trypsin for 45 min on ice. Internalized VHH was immunolabelled with an anti-myc tag and D-α-M-IR800 antibodies. Quantification of fluorescence is described in experimental procedures. Fluorescence intensities of the internalized amount is divided over the amount of cells (To-pro3 nuclear stain) and plotted against time. The signal of the irrelevant anti-hTfR VHH was deducted from the specific VHH signal and therefore represents the x-axis (n = 12) (s.e.m.'s are shown for all points).

Bivalent and biparatopic constructs were thus made using different linker lengths (5, 15 and 25 gly_4_-ser_1_ repeats) to account for possible preferred receptor orientations. An internalization assay as in fig. 2B was done to screen these constructs for internalization efficiency. For all bivalent constructs a slight but significant increase in internalization rate can be seen as compared with the respective monovalent constructs (fig. 4A), suggesting that the increase in endocytic rate is achieved via the dimerization of the receptors. No significant differences were observed between the different linker lengths. For the biparatopic constructs, a range of different internalization rates can be seen (fig. 4B). The slowest of which overlapped with the internalization speed of the bivalent constructs (e.g. 1d2-15-4b7, fig. 4C), but the fastest rates outpaced the bivalent constructs significantly (e.g. 4b11-5-1e7, fig. 4D). This effect cannot be solely due to the affinity of the constructs, because affinity determination on cells of several bivalent and biparatopic constructs indicated that the affinity of the biparatopics, although higher than monovalent constructs, did not exceed the affinity of the bivalent VHHs (fig. 1F), which suggests that the effect of the enhanced internalization rate is based on the endocytosis event. Combined, we show that internalization rates can indeed be improved by using bivalent and biparatopic constructs, possibly through the clustering of the receptor.

**Figure 5:**
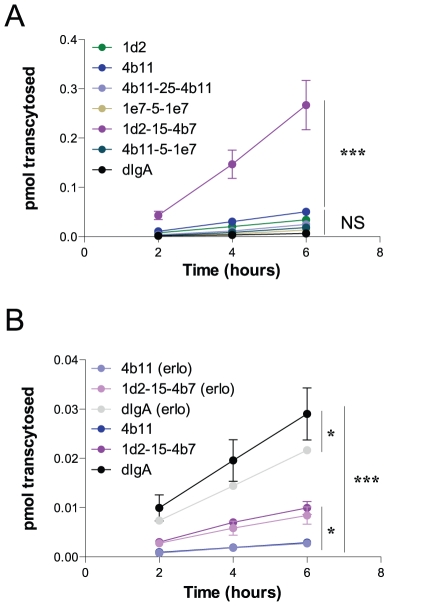
Transcytotic capacity of optimized constructs. (A+B) 1 nM ^125^I radio-labelled VHH or dIgA was applied in the basolateral chamber of polarized MDCK-hpIgR cells (A) or Caco-2 cells (B) grown on transwell inserts. Apical medium was resorbed after each 2 h interval to a maximum of 6 h and quantified separately. Amount of pmol that is transcytosed is plotted against time. Leakage of a non-specific VHH control was deducted from the specific signal (n = 4) (s.e.m.'s are shown for all points). Significance measurements were done by two-way ANOVA.

To see whether an increased endocytic rate also correlated with a higher transcytotic rate, 2 monovalent, 2 bivalent, 2 biparatopic constructs and dIgA were compared for their transcytotic capacity in a transwell assay. Constructs were radio-iodinated and probed basolaterally in a 6 hour time course. The monovalent, bivalent and one biparatopic construct all grouped together in a statistical insignificantly differing group (fig. 5A). The physiological ligand, dIgA, also had a similar transcytotic rate, which in literature has been suggested to be a non-enhanced steady-state transcytosis event in this model system [21]. However, one biparatopic construct, 1d2-15-4b7, had an enhanced transcytotic capacity as compared with all other constructs, transcytosing 5.6× more efficiently than the best monovalent construct (4b11) and 40× better than the ligand, dIgA. This feature was also checked in a Caco-2 cell system, which is a human intestinal cell line harbouring endogenous expression of the pIgR. Expression levels of hpIgR in Caco-2 are however a lot lower than in the transduced MDCK system. Accordingly, transcytosed amounts are significantly lower than the transcytotic capacities seen in the MDCK cell system. In the Caco-2 cell system the physiological ligand did show an enhanced transcytosis as compared with monovalent VHH transcytosis (10 fold), which is not totally in correspondence with what has been suggested in literature (compare [21] and [22], [23]). This transport was found to be partly dependent on EGFR kinase activity as has been described [24] (fig. 5B). In the Caco-2 system 1d2-15-4b7 also showed to be more effective in transcytosing to the apical side as compared with monovalent VHH (3.5 fold), however could not reach the same levels as the physiological ligand. Both the monovalent and the biparatopic transcytotic rates were hardly affected by addition of erlotinib (an EGFR kinase inhibitor), suggesting that the pathways taken by these VHHs reflect steady-state pIgR transcytosis and that the increase in transcytotic rate of 1d2-15-4b7 is solely determined by the increased endocytic rate seen in fig. 4C. These results show that enhancement of endocytosis and transcytosis can be achieved by using biparatopic VHH constructs.

## Discussion

The aim of this study was to select single domain llama antibody fragments that could exploit the pIgR transcytotic pathway. Monovalent constructs were isolated from phage display libraries and were found to transcytose across polarized epithelial monolayers and to be able to take along large particles of cargo. Biparatopic VHHs were constructed to optimize the transport and were found to transcytose more efficiently over an epithelial cell layer than the monovalent constructs. The ability of biparatopic VHHs to use the hpIgR to excrete 3.5 times more efficiently than the monovalent constructs makes them a very promising tool for the removal of unwanted agents, like viruses, toxins and other pathogens.

Previously, groups have shown that antibody fragments [23] and small peptides [15] selected to bind to the pIgR are able to use this receptor to enter cells and transcytose from the basolateral to the apical side. Here, we show a similar mechanism but with a different approach, namely with VHH technology. By making use of the easy genetic manipulation of the VHH genes, we were able to show that bivalent and biparatopic constructs were able to enhance the endocytosis of the hpIgR significantly, suggesting that these VHHs cluster the receptor and thereby speed up internalization. However, only one VHH of a selected few showed that increased endocytosis also correlated with a higher transcytotic capacity (1d2-15-4b7). This indicates that the system can be delicate, as increased internalization not always leads to an enhanced transcytotic capacity (e.g. 4b11-5-1e7). A possible explanation could be that on the one hand clustering can result in a heightened transcytosis rate but on the other hand can also target the receptor towards another fate (e.g. degradation in the lysosome or recycling back to the basolateral plasma membrane). This probably depends on the way the receptors are clustered (i.e. which epitope groups are linked). In this respect, the clustering should not be restricted to the use of biparatopic constructs but should also be possible with bivalent constructs. Screening of more bivalent and biparatopic compounds might therefore yield other VHHs that give similar results as 1d2-15-4b7.

The exact mechanism by which bivalent and biparatopic constructs enhance the internalization rate is not clear. Although rate enhancement through receptor clustering is a tempting mechanism of action, other effects are not excluded by these experiments. Cell binding of VHHs at a saturated (1 µM) concentration in the internalization assays might lead to binding of the biparatopic constructs to the cells with only one head. This can theoretically mean that two constructs can bind the receptor at the same time. Subsequent internalization would lead to two fold more construct entering cells than the monovalent constructs per time unit, thereby explaining why biparatopics give more signal than monovalent constructs in the internalization assay. The transcytosis assay, where a sub-saturated concentration was used (1 nM), showed that the capacity of 1d2-15-4b7 to cross epithelial monolayers is higher in both cell systems than the two fold increase we would expect in this scenario, suggesting that another mechanism must be at work. However, it is clear that more experiments will need to be done to understand the exact mode of action.

In literature it has been shown that human dIgA is not able to enhance transcytosis via signal transduction when interacting with the human pIgR in MDCK cells [21]. In this same paper, the authors show that this also holds true for a human lung epithelial cell carcinoma system having endogenous expression of the pIgR (Calu-3). This in contrast to studies done in MDCK cells with rabbit pIgR where they find that application of the human ligand to cells expressing rpIgR indeed elicits a signal transduction cascade which culminates in the enhancement of the transcytotic pathway [22]; an effect which was not seen when Fabs targeting the receptor were applied basolaterally [23]. Here, we find that human dIgA is indeed not able to transcytose faster than monovalent antibody fragments in the MDCK-hpIgR cell system. However, when applied to our human intestinal cell system harbouring endogenous expression of the hpIgR, we do see an enhancement of transcytosis when the ligand is applied basolaterally; an effect which is EGFR kinase activity dependent as is described for the MDCK-rpIgR cell system [24]. There are two possible explanations for these discrepancies. Either 1) the pIgR transcytotic system is differentially regulated in the human body depending on the epithelial tissue (enhancement in the intestines, but not the lungs) or 2) the genotype of either the Calu-3 or Caco-2 carcinoma cell line is altered as compared with the physiological situation in such a fashion that the cells have a lack or gain of expression of (a) certain gene(s) necessary to perform the transcytotic enhancement. *In vivo* or primary cell based experiments will need to be done to confirm this effect, however going by the MDCK-rpIgR and Caco-2 studies and the observation that early signal transduction mediators are activated in Calu-3 cells after pIgA stimulation, it seems safe to assume that the transcytotic enhancement effect based on signal transduction does occur in normal physiology.

The >10 fold higher affinity of the VHHs as compared to the physiological ligand [25] and the enhanced internalization of the bivalent constructs by receptor clustering make our VHH constructs attractive targeting modules for the export of unwanted molecules from the body. Currently, we are setting up *in vivo* experiments to confirm the ability of these constructs to serve as a carrier for removal of viruses by using multi-specific VHHs harbouring anti-hpIgR and anti-virus targeting heads. However, these first *in vitro* results are promising. They indicate that the selected constructs are able to transport large particles of cargo across artificial epithelial layers and that this transport can be enhanced 3–4 fold when using biparatopic constructs.

## Materials and Methods

### Immunization, selection and VHH production

Two *Llama glama's* were immunized with recombinant human pIgR ectodomain (R&D systems, Abingdon, UK) and subsequently boosted five times (protocol as described in [26]). The immune response of the animals was monitored by titration of blood samples on coated hpIgR wells. Four days after the last boost, lymphocytes were purified from a 200 ml blood sample by centrifugation on a Ficoll discontinuous gradient (Pharmacia, Uppsala, Sweden). RNA was subsequently extracted from these cells using guanidium thiocyanate [27] and a phage display library was prepared as has been described [28].

Phages displaying VHHs were produced by infecting phagemid transformed TG-1 bacteria with helper-phage VCS-M13 (Pharmacia, Uppsala, Sweden). The polyclonal phage pool was used for selections on coated plates. Phages were put on hpIgR coated plates (5, 0.5, 0 ug/ml) for 1 h shaking at room temperature. Plates were subsequently washed 20 times with PBS 0.05% tween and incubated with either 1 mg/ml trypsin for 20 min at room temperature or 500 µg/ml IgA (Sigma-Aldrich, Zwijndrecht, The Netherlands) for 4 h at room temperature. Trypsin activity was stopped after the incubation period with 200 µM ABSF. The solution now present in the wells was used to infect TG-1 bacteria for rescue.

Bivalent and biparatopic constructs were made using 5, 15 or 25 glycine serine linkers between both heads. Constructs were transformed in TG-1 bacteria and their production was induced with 0.1 mM IPTG when bacteria reached log-phase. After production, bacteria were frozen to release the periplasmic fraction. Purification of the periplasmic VHHs was done with the his-tag affinity matrix Talon (Clontech, St. Germain-en-Laye, France) and eluted with 150 mM Imidazol.

### Cell culture

MDCK type II (a kind gift of Dr. E. Regan-Klapisz, Utrecht University) were transduced with MLV harbouring a retroviral vector (pMX-IRES-Zeo) containing either nothing (‘MDCK control’) or the human pIgR cDNA (‘MDCK-hpIgR’) (the cDNA was a kind gift of Prof. Dr. C.S. Kaetzel, University of Kentucky). Virus was produced by transfecting the retroviral vector in HEK-ϕnx-cells with calcium-phosphate. These cells are themselves stably transfected with genes necessary for virus production. Virus was subsequently harvested the following days and put on MDCK cells which were transiently transfected with the murine ecotrophic receptor (which is necessary for virus infection). Cells were put on 250 µg/ml zeocin to select for transfected cells and subsequently FACS sorted to give a cell population with homogenous expression of hpIgR. Caco-2 cells were obtained from ATCC (ATCC number = HTB-37; Wesel, Germany).

Cells were cultured in DMEM supplemented with 8.2% FCS, 1× penicillin/streptomycin (P/S) and 1× L-glutamine (DMEM^+++^; Invitrogen, Breda, The Netherlands) and kept in a humidified, 5% CO_2_ stove at 37°C. Cells were passed on average three times/week with trypsin (PAA, Cölbe, Germany).

For polarized epithelial monolayers, 3×10^5^ cells were seeded per transwell filter (Corning, prod. n°: 3493, Amsterdam, The Netherlands) and cultured in DMEM-8.2% FCS-1× L-glut for 5 days (MDCK) or 21 days (Caco-2). Medium was refreshed every other day. The day before the experiment, monolayer integrity was checked with lucifer yellow (LY, Invitrogen, Breda, The Netherlands). Briefly, 25 µg/ml LY in DMEM-8.2% FCS-1× L-glut was added apically. A sample of the basolateral medium was taken at t = 0 and t = 1 h and quantitated with a FLUOstar system (BMG labtech, Isogen Lifescience, De Meern, The Netherlands). P_app_ was subsequently calculated using the formula described here [29]. When P_app_<1×10^−6^ (MDCK) or P_app_<5×10^−7^ (Caco-2), cells were assumed to have formed a tight, differentiated monolayer. Cells were put on CO_2_ independent medium (+8.2% FCS, penicillin/streptomycin and L-glut; Invitrogen, Breda, The Netherlands) after the measurement and put back into the stove overnight. P/S was added for the radio-active transcytosis and immunofluoresence assays, however was omitted for the phage-based assays.

### Pull down

1.8×10^6^ MDCK-hpIgR or MDCK-control cells were seeded the day before the experiment in a 6 wells plate. On the day of the experiment cells were put on ice, washed once with PBS and subsequently scraped with 40 µl/well RIPA buffer (20 mM Tris/HCl pH 7.5; 150 mM NaCl; 1 mM EDTA; 0.1% SDS; 0.5% Triton X-100). The buffer was resorbed from the wells plate, put in a reaction tube along with an extra 160 µl/well RIPA buffer and incubated for 5 min on ice. The lysate was centrifugated for 5 min at 14.000 rpm to remove cell debris. The supernatant was used as a source of cell-derived hpIgR. Medium that was cultured for three days on polarized MDCK-hpIgR cells was used as a source of hSC. The medium was resorbed and dialysed against PBS with a 6–8 kD MWCO membrane (Interchim, Montluçon, France) overnight at 4°C.

100 µl of the MDCK-hpIgR lysate or 4 µg sIgA (Sigma-Aldrich, Zwijndrecht, The Netherlands) was incubated with 10 µg VHH, 20 µl 1∶1 talon (Clontech, St. Germain-en-Laye, France) and 4 ml PBS for 1.5 h at 4°C head-over-head. The same amount of VHH and Talon was added to the hSC containing solution described above for secretory component precipitation. Beads were precipitated by centrifugation and washed once with 5 ml PBS and once with 1 ml PBS. 30 µl sample buffer was added, boiled at 100°C and separated with SDS-PAGE. The gel was blotted and the membrane was immunolabeled with M-α-Myc (9e10, courtesy of Dr. R.C. Roovers, Utrecht University), G-α-Secretory Component & G-α-IgA heavy chain (Sigma-Aldrich, Zwijndrecht, The Netherlands), R-α-MPO & D-α-GPO (Invitrogen, Breda, The Netherlands).

### Immunofluorescence

Cells were grown in transwell filters as described in the ‘Cell Culture’ section. Cells were blocked with 1% Marvel DMEM^+++^ for 15 min. VHHs were diluted to 1 µM in 1% Marvel-DMEM^+++^ and put on the cells for either 20 min (basolateral entry) or 1 h (apical entry) at 37°C. Cells were put on ice, washed twice with cold PBS and either directly fixed in 4% PFA for 15 min (basolateral entry) or first stripped with 25 µg/ml trypsin in DMEM for 45 min on ice and then fixed (apical entry). After fixation, cells were quenched at room temperature with 50 mM NH_4_Cl-PBS for 10 min and subsequently stained with M-α-Myc (4A6, Millipore, Amsterdam ZO, The Netherlands), R-α-VHH (in-house made), M-α-EEA1 (BD Biosciences, Breda, The Netherlands), G-α-R-A555, G-α-M-A488 (Invitrogen, Breda, The Netherlands) and DAPI (Roche, Almere, The Netherlands) in PBS-0.1% BSA-0.25% saponin. Filters are finally cut out, mounted with Slowfade (Invitrogen, Breda, The Netherlands) and imaged with a Zeiss LSM510 confocal laser scanning microscope.

### dIgA purification and labelling

dIgA was obtained by purifying IgA from human plasma (a kind gift of Dr. J.C. Stam, Utrecht University) using a CaptureSelect IgA column (BAC, Leiden, The Netherlands) according to the manufacturers protocol. The resulting IgA pool (both monomer as dimer) was labelled with NHS-IR800CW (Li-Cor, Westburg, Leusden, The Netherlands) in a protein∶dye molar ratio of 1∶4.1 and subsequently dialysed against PBS using 20 ml spin-columns (MWCO = 50.000 kD; Corning, Amsterdam, The Netherlands) to get rid of unlabelled dye and used for competition assays. The labelled protein pool was able to bind MDCK-hpIgR cells but not MDCK control cells, indicating that only the labelled dIgA was able to bind cells and not mIgA.

For radioactive transcytosis assays, the pool was further purified using a Superdex 200 gel permeation column (GE Healthcare, Hoevelaken, The Netherlands) thereby getting rid of more monomeric IgA. This to ensure that iodine labelling was done primarily on dIgA and that the bulk of protein loaded into the basolateral chamber was hpIgR reactive.

### Binding and competition assays

1.5×10^6^ cells were seeded the day before experimentation in a 96 wells plate. Cells were blocked with 1% Marvel-DMEM^+++^ for 15 min at 37°C and subsequently cooled to 4°C for 15 min. VHHs (either alone, with dIgA-IR800 or together) were diluted in DMEM-1% Marvel and cooled at 4°C for 15 min. VHHs were incubated for 1.5 h at 4°C, washed twice with ice-cold PBS, fixed for 15 min with 4% PFA on ice and quenched with 50 mM NH_4_Cl-PBS at room temperature for 10 min. VHHs were immunolabelled with M-α-Myc (4A6, Millipore, Amsterdam ZO, The Netherlands) and D-α-M-IR800 (Li-Cor, Westburg, Leusden, The Netherlands) in PBS-0.1% BSA and quantitated with an Odyssey imaging system (Li-Cor, Westburg, Leusden, The Netherlands). No further labelling was done after fixation in the competition assays using dIgA-IR800. Plotting was done with the ‘one-site specific binding’ algorithm in Graphpad Prism 5 (Graphpad Software Inc.).

### Internalization assay

1.5×10^6^ cells were seeded the day before experimentation in a 96 wells plate. Cells were blocked with 1% Marvel-DMEM^+++^ for 15 min at 37°C. VHHs were diluted to 1 µM in DMEM-1% Marvel and pre-heated to 37°C for 15 min. VHHs were incubated for indicated time points on the cells at 37°C. Cells were subsequently put on ice, washed twice with ice-cold PBS, stripped with 25 µg/ml trypsin in DMEM for 45 min on ice, washed once with PBS, fixed for 15 min with 4% PFA on ice and quenched for 10 min with 50 mM NH_4_Cl-PBS at room temperature. VHHs were subsequently immunolabelled with M-α-Myc (4A6, Millipore, Amsterdam ZO, The Netherlands), D-α-M-IR800 (Li-Cor, Westburg, Leusden, The Netherlands) and To-Pro-3 (Invitrogen, Breda, The Netherlands) in PBS-0.1% BSA-0.25% saponin and quantitated with an Odyssey imaging system (Li-Cor, Westburg, Leusden, The Netherlands).

### Radio-iodination

50 µg VHH or 20 µg dIgA was incubated with 0.2 mCi Na^125^I dissolved in a 0.5 M KPO_4_ buffer pH 7.5 in a tube which was coated with 0.1 mg Iodo-gen (Sigma-Aldrich, Zwijndrecht, The Netherlands) for approximately 10 min. The reaction was stopped by addition of an excess 0.25 M KPO_4_ pH 7.5-1% BSA and the mixture was subsequently put on an Ag-1-8× resin column (Bio-Rad, Veenendaal, The Netherlands) to separate free from labelled radio-active dye. The percentage free dye was subsequently determined by TCA precipitation and did not exceed 8% of total amount of counts. A binding assay showed that most VHHs are unaffected by the labelling. However, bivalent constructs harbouring VHH 1e7 did show some lack of binding.

### Transcytosis assay

On the day of the experiment, cells were blocked in 1% casein in CO_2_ independent medium (+8.2% FCS, L-glut and either with or without P/S depending on whether phages were used in the assay) for 15 min at 37°C. Phages were diluted in the same blocking buffer so that there were 1.67×10^9^ phages in 500 µl. This mixture was incubated for 5 h. Medium of the opposing chamber was resorbed and 100 µl (1/3 of total volume) of this output was mixed with log-phase TG-1 for 45 min at 37°C for infection. Bacteria were subsequently put on ampicillin containing agar-plates and grown overnight.

For iodinated transcytosis assays, cells were blocked in the same fashion as above and 1 nM of radio-iodinated VHH/dIgA was added to the basolateral chamber. 10 µM erlotinib or 0.1% DMSO (Sigma-Aldrich, Zwijndrecht, The Netherlands) was added 30 min before the start of the experiment and was present during the whole time course. The apical medium was resorbed after each two hour period up till a maximum duration of 6 h for the experiment. Apical media were quantitated with a Perkin Elmer Wizard counter (Groningen, The Netherlands).

## Supporting Information

Figure S1 **Colocalization study of internalized VHH 4b7 with endosomal marker EEA1.** 1 µM VHH was applied either apically for 1 h or basolaterally for 20 min. Cells were fixed and immunolabelled with R-α-VHH (Alexa-555) and M-α-EEA1 (Alexa-488) and stained with DAPI.
(EPS)

Figure S2 **Epitope mapping of VHHs.** 30 nM of myc-VHH is incubated with indicated amounts of flag-VHH in an hpIgR coated well for 2 h at room temperature. Myc tag presence is subsequently quantified and plotted against the concentration of flag-VHH (n = 2).
(EPS)
